# Outcomes of allogeneic haematopoietic stem cell transplantation with intensity-modulated total body irradiation by helical tomotherapy: a 2-year prospective follow-up study

**DOI:** 10.1080/07853890.2022.2125171

**Published:** 2022-10-17

**Authors:** Tatsuya Konishi, Hiroaki Ogawa, Yuho Najima, Shinpei Hashimoto, Satoshi Kito, Yuya Atsuta, Atsushi Wada, Hiroto Adachi, Ryosuke Konuma, Yuya Kishida, Akihito Nagata, Yuta Yamada, Satoshi Kaito, Junichi Mukae, Atsushi Marumo, Yuma Noguchi, Naoki Shingai, Takashi Toya, Aiko Igarashi, Hiroaki Shimizu, Takeshi Kobayashi, Kazuteru Ohashi, Noriko Doki, Keiko Nemoto Murofushi

**Affiliations:** a Hematology Division, Tokyo Metropolitan Cancer and Infectious Diseases Center, Komagome Hospital, Tokyo, Japan; b Department of Radiology, Division of Radiation Oncology, Tokyo Metropolitan Cancer and Infectious Diseases Center, Komagome Hospital, Tokyo, Japan

**Keywords:** Intensity-modulated radiation therapy, allogeneic stem cell transplantation, total body irradiation, helical tomotherapy

## Abstract

**Background and objectives:**

Intensity-modulated radiation therapy (IMRT) helps achieve good radiation dose conformity and precise dose evaluation. We conducted a single-centre prospective study to assess the safety and feasibility of total body irradiation with IMRT (IMRT-TBI) using helical tomotherapy in allogeneic haematopoietic stem cell transplantation (allo-HSCT).

**Patients and methods:**

Thirty-nine adult patients with haematological malignancy (acute lymphoblastic leukaemia [*n* = 21], chronic myeloid leukaemia [*n* = 6], mixed phenotype acute leukaemia [*n* = 5], acute myeloid leukaemia [*n* = 4], and malignant lymphoma [*n* = 3]) who received 12 Gy IMRT-TBI were enrolled with a median follow-up of 934.5 (range, 617–1254) d. At the time of transplantation, 33 patients (85%) achieved complete remission. The conditioning regimen used IMRT-TBI (12 Gy in 6 fractions twice daily, for 3 d) and cyclophosphamide (60 mg/kg/d, for 2 d), seven patients were combined with cytarabine, and five with etoposide. We set dose constraints for the lungs, kidneys and lens as the organs at risk.

**Results:**

The mean doses for the lungs and kidneys were 7.50 and 9.11 Gy, respectively. The mean maximum dose for the lens (right/left) was 5.75/5.87 Gy. The 2-year overall survival (OS), disease-free survival (DFS), cumulative incidence of relapse (CIR) and non-relapse mortality (NRM) were 69, 64, 18 and 18%, respectively. Thirty-six patients developed early adverse events (AEs) (including four patients with Grade 3/4 toxicities), most of which were reversible oral mucositis and may partially have been related to IMRT-TBI. However, the incidence of toxicity was comparable to conventional TBI-based conditioning transplantation. None of the patients developed primary graft failure, or Grade III–IV acute graft-versus-host disease (GVHD). In late complications, chronic kidney disease was observed in six patients, a lower incidence compared to conventional TBI-based conditioning transplantation. No radiation pneumonitis or cataracts were observed in any of the patients.

**Conclusions:**

IMRT-TBI is safe and feasible for haematological malignancies with acceptable clinical outcomes.

**KEY MESSAGES**
1. IMRT-TBI-helical tomotherapy aids in accurate dose calculation and conformity.
2. It could be used without any considerable increase in the rate of TBI-related AEs.
3. Allo-HSCT with IMRT-TBI may be an alternative to conventional TBI for clinical use.

## Introduction

Total body irradiation (TBI) is widely used as part of a conditioning regimen in allogeneic haematopoietic stem cell transplantation (allo-HSCT) [1]. Conventional TBI methods, such as the moving couch or long source-to-skin distance using lead blocks, have been established over the last three decades. However, these TBI methods are associated with a higher risk of adverse events (AEs), some of which are treatable, while others may progress to long-term or permanent sequelae associated with high non-relapse mortality (NRM) rates [2–7]. Conventional TBI does not allow for accurate calculations of doses to targets and organs at risk, such as the lung, kidney and lens. Therefore, it is difficult to reduce the rates of AEs while maintaining sufficient antitumor effects after conventional TBI.

Intensity-modulated radiation therapy (IMRT) is an advanced radiotherapy technique with high conformity of radiation dose distribution to the target volume while sparing adjacent normal tissue [8]. Recent studies have shown the clinical feasibility of TBI with IMRT (IMRT-TBI) to help avoid severe AEs or increased cumulative incidence of relapse (CIR) [9,10]. However, few studies have investigated the long-term clinical outcomes and safety of IMRT-TBI in allo-HSCT recipients.

We have previously reported on the short-term safety of IMRT-TBI using helical tomotherapy in 10 allo-HSCT recipients included in the first prospective clinical study in Japan [11]. This primary study found that IMRT-TBI-based conditioning was well-tolerated as regards early AEs in adult allo-HSCT recipients with haematological malignancy in complete remission. This study was designed as a single-centre prospective study of IMRT-TBI-based conditioning in allo-HSCT recipients, which aimed to examine the safety and feasibility of IMRT-TBI using helical tomotherapy with a 2-year follow-up.

## Material and methods

### Patients

Our study aimed to examine in detail the safety and feasibility of IMRT-TBI in patients with haematological malignancies. We enrolled patients aged from 18 to 60 years who were planning to receive 12 Gy TBI as a myeloablative conditioning regimen prior to allo-HSCT at our hospital. The inclusion criteria were as follows: (1) an Eastern Cooperative Oncology Group performance status of 0–2; (2) left ventricular cardiac ejection fraction of ≥50%; (3) forced vital capacity of ≥70% and forced expiratory volume in 1 s of ≥70%; (4) serum total bilirubin levels of ≤2 mg/dL, alanine aminotransferase and aspartate aminotransferase levels exceeding the upper limit of the normal range by up to 5-fold; and (5) calculated creatinine clearance rate of ≥30 mL/min/m^2^. The exclusion criteria were as follows: (1) uncontrolled extramedullary disease at the time of transplantation; (2) a history of allo-HSCT or autologous transplantation; (3) other malignancies; (4) uncontrolled diabetes mellitus; and (5) pregnancy. All patients were evaluated at a multidisciplinary transplant decision meeting to determine if they were eligible for transplantation. Transplant-eligible patients were enrolled in this study even if they were not in remission. This study was approved by the Institutional Review Board of the Tokyo Metropolitan Cancer and Infectious Diseases Centre, Komagome Hospital (reference number 2138), and was registered in UMIN Clinical Trials Registry (UMIN-CTR: 000033248). Written informed consent was obtained from all participants, and the study was conducted in accordance with the principles of the Declaration of Helsinki.

### IMRT-TBI procedures

The irradiation procedures followed in this study have been previously described [11]. The TomoTherapy Radixact^TM^ (Accuray, Inc., Sunnyvale, CA), a new generation of helical tomotherapy platform, was used for IMRT-TBI. The prescribed dose for TBI was 12 Gy in 6 fractions, twice daily for 3 consecutive days, and the interval between each fraction was >6 h. The date of IMRT-TBI was scheduled before or after chemotherapy depending on the available date for irradiation at our hospital and its coordination with the transplantation date. Computed tomography images with a slice thickness of 5 mm were obtained to plan treatment approximately 2 weeks before IMRT-TBI. The lungs, kidneys, and lens were regarded as organs at risk because they are considered for protection in conventional TBI. The clinical target volume was defined as the whole body without the organs at risk, which was equivalent to the planning target volume (PTV). For the PTV, 80% of the minimum (D_80%_) and maximum (D_max_) doses were set to 98–105% and 115% of the prescribed doses, respectively. Based on the conventional TBI methods in our hospital, the dose constraints for the organs at risk were set as follows: the average dose was <8 Gy, and the minimum dose received by 2% (D_2%_) of the organ was less than 12 Gy in the lungs; the average dose was less than 10 Gy, and D_2%_ was less than 12 Gy in the kidneys; finally, the average dose was less than 6 Gy and D_max_ was less than 10 Gy in the lens. Given the maximum moving capacity of the couch in the Radixact^TM^, the radiation field was divided into two parts, namely, the head and foot. Dosimetric verification of the treatment plan was conducted as per the physical technology guidelines of IMRT recommended by the Japanese Society of Radiation Oncology [12].

### Other transplantation procedures

The standard conditioning regimen consisted of IMRT-TBI (12 Gy in 6 fractions twice daily, 3 d) and cyclophosphamide (CY, 60 mg/kg/d, 2 d). Several previous studies showed an enhanced antitumor efficacy with the addition of etoposide or cytarabine to CY for advanced-risk lymphoid tumours (positive minimal residual disease, presence of poor-risk cytogenetics, an initial elevated leukocyte count, or beyond CR2) or the addition of cytarabine for myeloid tumours with high-risk (based on the disease type and genetic mutations) or cord blood transplantation [13–16]. In these studies, the attending physician evaluated the patient based on disease risk and transplant settings, and additional therapy was allowed as needed. Standard graft-*versus*-host disease (GVHD) prophylaxis consisted of cyclosporine or tacrolimus and short-term methotrexate or mycophenolate mofetil. Anti-thymocyte globulin (Thymoglobulin, Sanofi, Tokyo, Japan) was used for patients with a high risk of GVHD, which was determined by physicians following an evaluation [17]. Granulocyte-colony stimulating factor was started on day 5 after transplantation. Neutrophil engraftment was defined as the first of 3 consecutive days with an absolute neutrophil count of >0.5 × 10^9^/L. Platelet engraftment was defined as the first of 7 consecutive days with the platelet count of >20 × 10^9^/L without transfusion support. Prophylaxis for bacterial and fungal infections consisted of oral quinolones and oral fluconazole or itraconazole. Oral acyclovir was used as prophylaxis for herpes virus infection. For patients at high risk of cytomegalovirus reactivation, letermovir was used as primary prophylaxis from transplantation until day 100. Acute and chronic GVHD (aGVHD and cGVHD, respectively) were diagnosed and graded using previously established criteria [18,19]. Human leukocyte antigen disparities were categorized as GVHD or rejection direction.

### Adverse events evaluation

AEs associated with regimen-related toxicity were evaluated according to the Bearman criteria [20]. Other AEs were graded based on the National Cancer Institute Common Terminology Criteria for Adverse Events version 4.0. Several haematologists and radiologists actively and prospectively monitored subjective symptoms and other findings after IMRT-TBI, and performed evaluations to determine whether complications were related to IMRT-TBI. Major radiation-related severe AEs, such as interstitial pneumonia (IP), sinusoidal obstruction syndrome (SOS) and renal dysfunction were carefully evaluated and classified into early and late complications. Complications such as cataracts were evaluated by a specialist for a definitive diagnosis. The regular evaluation included a daily check-up, blood tests at least once a week, and additional tests, such as imaging as required. After discharge, a routine medical check-up and blood tests were performed at least once a month for the first year after transplantation, as well as periodic respiratory or thyroid function tests to determine post-transplant toxicities even in the absence of symptoms.

### Statistical analysis

Overall survival (OS) was defined as the time from allo-HSCT to death due to any cause. Disease-free survival (DFS) was defined as the time from allo-HSCT to relapse or death. The probabilities of OS and DFS were estimated using the Kaplan–Meier product limit method and compared using the log-rank test. The rates of CIR, NRM and GVHD were evaluated using the Grey’s method, considering relapse as a competing risk factor for NRM, and death without relapse as a competing risk factor for CIR. All statistical tests were two-sided, and statistical significance was set at *p* < .05. All statistical analyses were performed using R Statistical Software version 4.1.1 (R Foundation for Statistical Computing, Vienna, Austria).

## Results

### Patient characteristics

Patient characteristics are summarized in Table 1. A total of 39 (19 males) were recruited between July 2018 and April 2020 and were followed up until 31 December 2021. The median follow-up duration for survivors was 934.5 (range, 617–1,254) d. The median age was 44 (range, 19–54) years. The underlying diagnoses were acute lymphoblastic leukaemia (*n* = 21), chronic myeloid leukaemia (*n* = 6), mixed phenotype acute leukaemia (*n* = 5), acute myeloid leukaemia (*n* = 4) and malignant lymphoma (*n* = 3) including peripheral T cell lymphoma (*n* = 2) and Hodgkin’s lymphoma (*n* = 1). Five patients had a history of extramedullary disease. Thirty-three patients (85%) underwent transplantation in remission, including seven in molecular remission. According to the refined disease risk index (R-DRI) [21], patients were classified as at low, intermediate, high, and very high risk in 7 (18%), 20 (51%), 8 (21%) and 4 (10%) cases, respectively. Ten patients underwent IMRT-TBI prior to CY administration, and the remaining patients received IMRT-TBI after CY. Six patients had non-complete remission at the time of allo-HSCT. In the conditioning regimen, chemotherapies with IMRT-TBI were CY (*n* = 27), cytarabine + CY (*n* = 7) and etoposide + CY (*n* = 5). Three patients received anti-thymocyte globulin-based GVHD prophylaxis.

**Table 1.** Patient characteristics.

Factor	Total
*N*	39
Median age at SCT, year (range)	44 (19–54)
Sex, male, *n* (%)	19 (48.7)
Disease, *n* (%)	
ALL	21 (53.8)
CML	6 (15.4)
MPAL	5 (12.8)
AML	4 (10.3)
ML	3 (7.7)
Disease status, *n* (%)	
CR or CP	33 (84.6)
Non-CR	6 (15.4)
R-DRI, *n* (%)	
Low	7 (17.9)
Intermediate	20 (51.3)
High	8 (20.5)
Very high	4 (10.3)
PS, *n* (%)	
0	32 (82.1)
≥1	7 (17.9)
HCT-CI, *n* (%)	
0	27 (69.2)
1	6 (15.4)
2	3 (7.7)
3	3 (7.7)
Donor, *n* (%)	
Unrelated	31 (79.5)
Related	8 (20.5)
HLA disparity, *n* (%)	
8/8 matched	24 (61.5)
7/8 matched	8 (20.5)
<7/8 matched	7 (17.9)
Stem cell source, *n* (%)	
Bone marrow	21 (53.8)
Peripheral blood	10 (25.6)
Cord blood	8 (20.5)
GVHD prophylaxis, *n* (%)	
FK + sMTX	28 (71.8)
CsA + sMTX	8 (20.5)
FK + MMF	3 (7.7)
Conditioning regimen, *n* (%)	
CY + TBI	27 (69.2)
CA + CY + TBI	7 (17.9)
VP-16 + CY + TBI	5 (12.8)

ALL: acute lymphoblastic leukaemia; AML: acute myeloid leukaemia; CA: cytarabine; CML: chronic myeloid leukaemia; CP: chronic phase; CR: complete remission; CsA: cyclosporine; CY: cyclophosphamide; FK: tacrolimus; GVHD: graft-versus-host disease; HCT-CI: haematopoietic cell transplantation-specific comorbidity index; HLA: human leukocyte antigen; ML: malignant lymphoma; MMF: mycophenolate mofetil; MPAL: mixed phenotype acute leukaemia; PS: performance status; R-DRI: refined disease risk index; SCT: stem cell transplantation; sMTX: short-term methotrexate; TBI: total body irradiation; VP-16: etoposide

### Radiation dose distributions

The radiation doses to the PTV and organs at risk are described in Table 2. The median D_80%_ of the PTV in all patients was 12.01 (range, 11.94–12.06) Gy. The average values of the mean radiation doses for the lungs and kidneys were 7.50 and 9.11 Gy, respectively. The mean D_max_ for the lens (right/left) was 5.75/5.87 Gy. The treatment plan for all patients met the dose constraints.

**Table 2.** Dose measurement results for all patients.

	PTV		Lungs		Kidneys		Lens (right)		Lens (left)	
	D_80%_	D_max_	Mean	D_max_	Mean	D_max_	D_80%_	D_max_	D_80%_	D_max_
Mean, Gy	12	14.7	7.5	12.54	9.11	12.25	3.9	5.75	3.99	5.87
Median, Gy	12.01	14.63	7.49	12.54	9.08	12.22	3.83	5.67	3.67	5.7

PTV: planning target volume

### Engraftment and clinical outcomes

**Figure 1.**
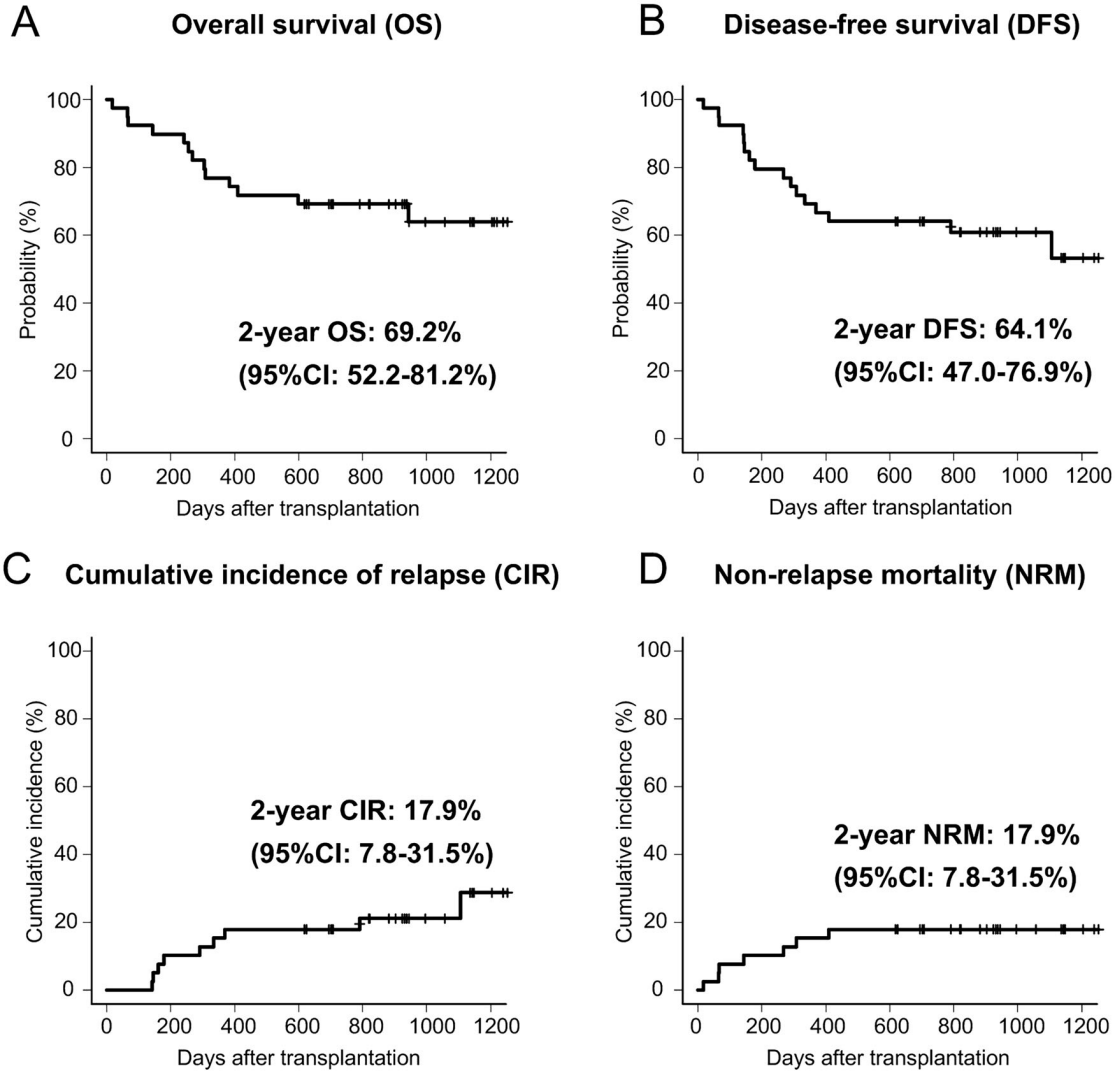
Overall survival (A), disease-free survival (B), cumulative incidence of relapse (C) and non-relapse mortality (D) in 39 patients who underwent allo-HSCT with IMRT-TBI.

**Figure 2.**
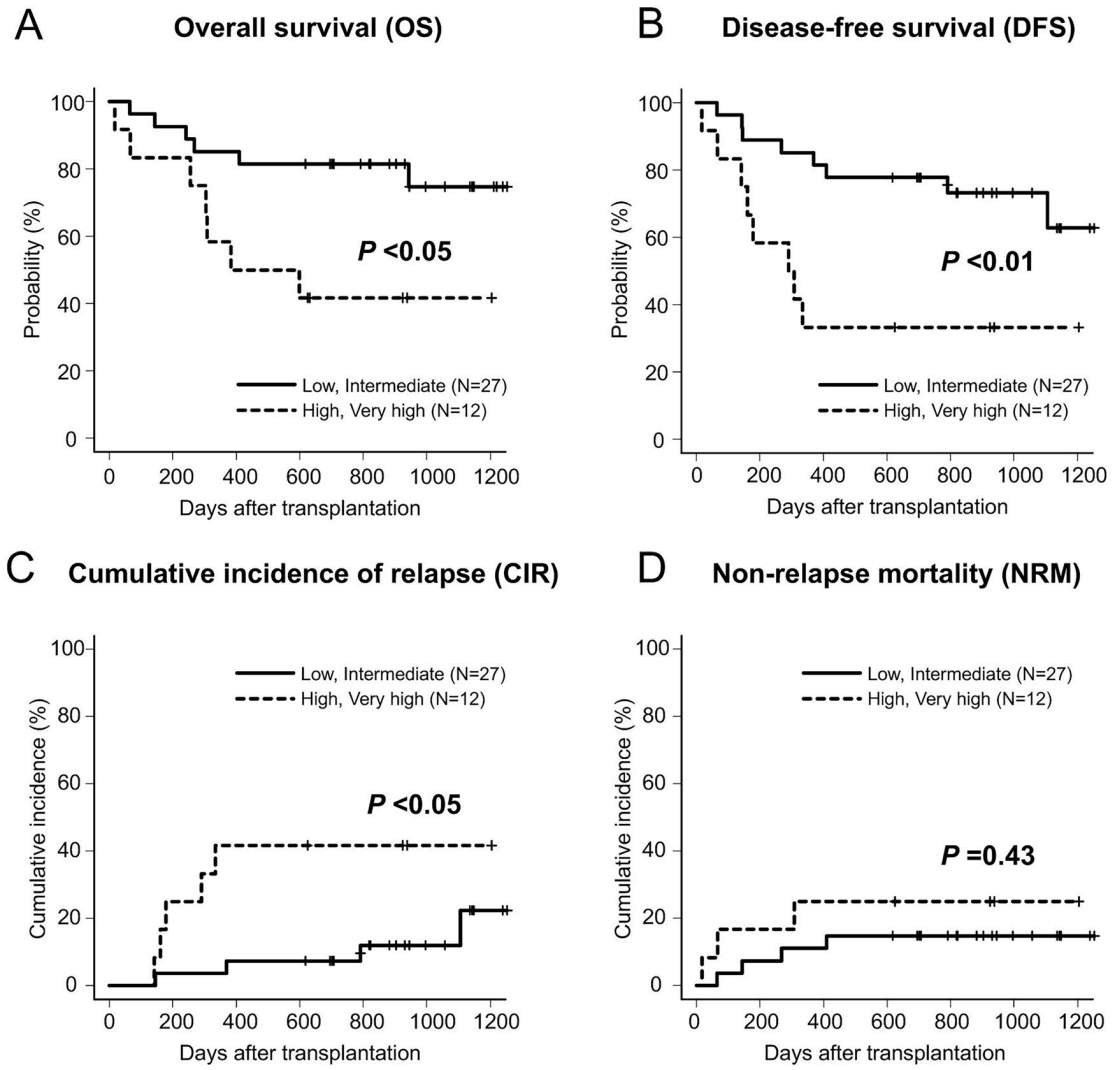
Overall survival (A), disease-free survival (B), cumulative incidence of relapse (C) and non-relapse mortality (D) in 39 patients who underwent allo-HSCT with IMRT-TBI, according to the refined disease risk index (low/intermediate, *n* = 27 vs. high/very high, *n* = 12).

All patients achieved neutrophil engraftment within a median duration of 19 (range, 12–33) days. Platelet engraftment was achieved in 32 (82%) patients within a median duration of 38 (range, 13–223) d. The 2-year OS and DFS rates for the entire cohort were 69% (95% confidence interval [CI]: 52–81%) and 64% (95% CI: 47–77%), respectively (Figure 1(A,B)). The 2-year CIR and NRM rates were 18% (95% CI: 8–32%) and 18% (95% CI: 8–32%), respectively (Figure 1(C,D)). According to R-DRI, the OS and DFS rates were significantly higher in the low or intermediate R-DRI group than in the high or very high R-DRI groups (82 vs. 42% at 2-year OS, *p* < .05; 78 vs. 33% at 2-year DFS, *p* < .01) (Figure 2(A,B)). The CIR was significantly lower in the low or intermediate R-DRI group than in the high or very high R-DRI group (7 vs. 42% at 2-year CIR, *p* < .05) (Figure 2(C)). By contrast, the NRM did not differ between the groups (15 vs. 25% at 2-year NRM, *p* = .43) (Figure 2(D)). Based on the conditional chemotherapies with IMRT-TBI, the OS rate for CY was not significantly different from that of cytarabine + CY (78 vs. 57% at 2-year OS, *p* = .27), but tended to be higher than that of etoposide + CY (78 vs. 40% at 2-year OS, *p* = .06). Thirteen (33%) patients had died at the time of the last follow-up due to infection (*n* = 6), disease relapse (*n* = 4), SOS (*n* = 2) and secondary graft failure (*n* = 1).

### Early AEs and aGVHD

Thirty-six (92%) patients developed early AEs within 100 days following allo-HSCT, including regimen-related toxicities and complications. Conditioning regimen-related toxicities are summarized according to the Bearman criteria in Table 3. The most frequent toxicity type was oral mucositis, which was observed in 25 (64%) patients, although none of the patients with oral mucositis developed toxicity more severe than Grade 2. Grade 3 or 4 toxicities were observed in four (10%) patients. Early complications, excluding regimen-related toxicities, were reported in 5% of the patients (Table 4). Positive cytomegalovirus antigenemia, the most common early complication, developed in 15 (39%) patients and no patients suffered from cytomegalovirus disease. Among these, acute kidney injury, SOS and graft failure were suspected of being partly caused by IMRT-TBI. However, there was no increase in incidence compared with the general incidence associated with other allo-HSCT using TBI (Supplementary Table 1). Within 100 d of allo-HSCT, three patients died without any signs of relapse. The causes of death were SOS (*n* = 2) and graft failure (*n* = 1). Among them, only one patient died within 28 d following allo-HSCT. This patient, who had a history of paroxysmal supraventricular tachycardia, was conditioned by etoposide + CY + IMRT-TBI and underwent bone marrow transplantation from an unrelated donor for acute lymphoblastic leukaemia in the second complete remission. After the transplant, he suffered from acute kidney injury (day 5) and refractory supraventricular tachycardia (Grade 3 on day 7 according to the Bearman criteria, which was finally graded as Grade 4 due to death), leading to unstable haemodynamics. Finally, the patient died of SOS on day 18. In addition, 25 (64%) patients developed Grade I to II aGVHD (Grade I, *n* = 12; Grade II, *n* = 13). Organ manifestations of aGVHD were the skin (*n* = 24), gut (*n* = 6) and liver (*n* = 1). None of the patients had Grade III–IV aGVHD.

**Table 3.** Adverse events according to the Bearman criteria.

Toxicity, *n*	Any grade	Grade 3	Grade 4
Heart	6	1	1
Bladder	4	0	0
Kidneys	19	2	0
Lungs	2	0	0
Liver	7	1	0
CNS	6	0	0
Mucosa	25	0	0
Gut	13	1	0

CNS: central nervous system

**Table 4.** Early complications (within 100 d after transplant).

	*n* (%)
CMV antigenemia	15 (38.5)
Bacteraemia	10 (25.6)
AKI	7 (17.9)
Haemorrhagic cystitis	4 (10.3)
HHV6 infection	3 (7.7)
SOS	3 (7.7)
HPS	3 (7.7)
Minor bleeding	2 (5.1)
CDI	2 (5.1)
Pneumonia	2 (5.1)
Graft failure	2 (5.1)
Engraftment syndrome	2 (5.1)

AKI: acute kidney injury; CDI: *Clostridium difficile* infection; CMV: cytomegalovirus; HHV6: human herpes virus 6; HPS: hemophagocytic syndromes; SOS: sinusoidal obstruction syndromes

### Late AEs and cGVHD

Two patients developed bacterial infections more than 100 d following allo-HSCT, and both cases followed a fatal course. One patient developed a brain abscess, and the other developed necrotizing fasciitis. Nine (23%) patients developed organ cGVHD by the time of the analysis. In addition, cGVHD affected the mouth (*n* = 6), skin (*n* = 3), joints and fascia (*n* = 2), eyes (*n* = 1) and liver (*n* = 1). Severe cGVHD occurred in only one patient. None of the patients developed lung or renal cGVHD. Chronic kidney disease (except GVHD) developed in six patients (15%) as a complication suspected of being partially related to IMRT-TBI. However, none of the patients required maintenance haemodialysis, neither were IP or cataracts detected during the observation period of this study.

## Discussion

We performed allo-HSCT with 12 Gy in 6 fractions twice daily for 3 consecutive days of IMRT-TBI using helical tomotherapy, and all patients were successfully irradiated without physical or technical problems. Although previous studies have assessed the clinical safety of IMRT-TBI [9,10], this relatively larger study evaluated the safety and feasibility of IMRT-TBI. Conventional irradiation planning of TBI using lead blocks for shielding is difficult to finely customize for each patient (Supplementary Figure 1(A)). This study demonstrated the safety and feasibility of IMRT-TBI using helical tomotherapy, which allows for accurate radiation dose calculation and dose conformity (Supplementary Figure 1(B)), and included the incidence of complications observed in the organs at risk (Tables 3 and 4). In addition, no patient developed primary graft failure or Grade III to IV aGVHD in this study.

Although the dominant early AEs in patients undergoing a TBI-based conditioning regimen are pain and nausea caused by oral mucositis, these symptoms are easily manageable with symptomatic drugs [22]. Similar gastrointestinal AEs were observed in the early phase of allo-HSCT with IMRT-TBI in this study. However, the mucosal injury did not become severe, and the observed AEs were treatable.

Supplementary Table 1 summarizes the clinical studies of conventional TBI and IMRT-TBI in which complications have been sufficiently evaluated with relatively long-term follow-up (more than 2 years) of a large number of patients (more than 10 patients for the IMRT study, and more than 100 patients for conventional TBI study) [23–31]. We set dose constraints for the lungs, kidneys and lens as the organs at risk for TBI-related AEs. Some reports of IP after TBI have shown a decrease in the rates of AEs when the total irradiation dose was reduced [32–34]. In a recent study of total marrow irradiation, a reduction in IP was observed when the irradiation dose for the lung was kept below 8 Gy [32]. In this study, IMRT-TBI allowed for accurate dose calculations; the average mean radiation dose for the lung was confirmed as lower than 8 Gy, and none of the patients developed IP or idiopathic pneumonia syndrome. In previous reports, the median times to IP onset and idiopathic pneumonia syndrome were 2.5 months (80% of patients onset less than 6 months) and 0.7 months after allo-HSCT containing TBI-based regimens, respectively [33,35]. In addition, a recent study showed the median onset of lung complications after TBI was 16 months [36]. The follow-up period in our study was more than 20 months for all patients (median, 30.7 months), which would cover the predominant onset time of radiation-related lung toxicities. TBI has been suggested as a possible cause of renal toxicity, also known as radiation nephropathy, although previous findings are inconsistent [4,37]. Chronic kidney disease is common in survivors of allo-HSCT, affecting 18–66% of adult patients [37]. In this study, the cumulative incidence of chronic kidney disease was 15%, which was lower than that previously reported. Cataract is one of the most common late complications following allo-HSCT. According to the long-term follow-up data in survivors of myeloablative conditioning allo-HSCT for acute myeloid leukaemia, high-dose TBI exposure and cGVHD were independently associated with increased risk of cataracts [5]. Proper shielding or the use of lower-dose fractionated TBI could decrease the incidence of cataracts [38]. The use of IMRT-TBI allowed for very low doses to the bilateral lens. Although the observation period was approximately 2 years, no patient developed cataracts in this study. The maximum dose-rate for TBI by helical tomotherapy is 1000 cGy/min, which is higher than that for conventional TBI. In this study, the average dose-rates calculated from the prescribed dose, the effective field width and couch movement time (1.0 mm/s for the head and trunk, 1.1 mm/s for the foot) were 133.0 cGy/min for the head and trunk, 146.3 cGy/min for the foot. Regarding concerns about dose-rate-related complications, higher dose-rate may be associated with increased lung or renal toxicities in conventional TBI [2,39–43]. However, there are no previous reports that show increasing lung and renal complications for IMRT-TBI and total marrow and lymphoid irradiation (TMLI) using helical tomotherapy. Overall, TBI-related AEs for each organ did not increase in allo-HSCT with IMRT-TBI by helical tomotherapy, compared with conventional TBI. In addition, the present evidence suggests that the precise dose evaluation and dose constraints for organs at risk using IMRT-TBI are sufficiently feasible to be compared with conventional TBI (Supplementary Table 1). These results may assist in preventing radiological AEs in allo-HSCT with TBI.

SOS is a life-threatening AE associated with allo-HSCT with and without TBI, and three (8%) patients developed SOS in this study. One patient was treated with inotuzumab ozogamisin, which is a risk factor for the development of SOS [44]. Moreover, all patients who developed SOS in this study received additional cytarabine or etoposide as conditioning chemotherapy with CY. The mean values of liver doses for three patients with SOS were 11.95, 12.18 and 12.20, which were not particularly higher than the median for all patients (median 12.13; range, 11.95–12.31). Notably, previous retrospective studies have shown that the overall mean incidence rate of SOS was 10.3 and 13.7% [45,46], suggesting no increase in its incidence when using IMRT-TBI.

In conditioning chemotherapy, the addition of cytarabine or etoposide to CY + IMRT-TBI to increase the antitumor effect has previously been examined in terms of efficacy and safety [13–16]. Nevertheless, the 2-year OS rate for CY (78%) tended to be higher than that for etoposide + CY (40%). However, it should be noted that additional antitumor agents are only used for patients with high-risk disease. Therefore, we evaluated clinical outcomes by classifying patients according to the R-DRI. As a result, the 2-year OS rate was 82% in the low/intermediate R-DRI group and 42% in the high/very high R-DRI group (Figure 2(A)), suggesting that there was no apparent negative impact on survival rates compared to those previously reported [21]. Establishing the optimal dose and combination chemotherapy requires further research.

For the patients with high disease risk, CIR reached over 40% (Figure 2(C)) which significantly reduced the OS of this population. To prevent the relapse after HSCT, recent studies have examined dose escalation using TMLI to further improve clinical outcomes [47,48]. Based on the results of this study confirming the safety and feasibility of IMRT-TBI 12 Gy, our institute is currently investigating the safety of TMLI with the dose escalation up to 18 Gy/6 Fr/3 d (UMIN-CTR: 000037581).

As a single-centre evaluation, this study has several limitations. There were multiple variations in the conditioning regimen, which made it difficult to judge whether an AE was caused by TBI or by another factor. In addition, the follow-up period was relatively short, precluding the assessment of late complications, such as cataracts or cGVHD. Further studies are needed to determine the optimal dose constraints for the organs at risk. Regarding the treatment time, each fractional treatment time (including patient immobilization, beam-on and frame rotation time) is approximately two to three times longer than that of conventional TBI, which is time-consuming for both the patient and technician. The time required for IMRT-TBI planning is also longer than for conventional TBI. However, IMRT-TBI has the ability to accurately assess radiation dose, which has the advantage of allowing future evaluations of the appropriate dose control to organs at risk, and their effect on long-term AEs. In addition, the implementation of new technologies, such as an artificial intelligence-assisted automatic contouring algorithm and rapid optimization algorithm of doses for target and organs at risk, are promising to reduce planning time in the future. Furthermore, we believe that the establishment of the TMLI method will provide sufficient benefit to the patient to surpass these concerns. The present findings may provide a foundation that can be applied by future dose escalation studies using TMLI.

In conclusion, we prospectively established that IMRT-TBI is feasible in adult patients who undergo allo-HSCT. The outcomes evaluated in this study suggest that allo-HSCT with IMRT-TBI may be a valuable alternative to conventional TBI in clinical practice. However, further research is required to assess the long-term outcomes of IMRT-TBI.

## Supplementary Material

Supplemental Material

## Data Availability

The datasets analysed during this study are available from the corresponding author upon reasonable request.
