# Supplementary material for: Outcomes of allogeneic haematopoietic stem cell transplantation with intensity-modulated total body irradiation by helical tomotherapy: a 2-year prospective follow-up study
Source: Ann Med. 2022 Oct 17;54(1):2617–26. doi: 10.1080/07853890.2022.2125171 (PMC9624256; doi:10.1080/07853890.2022.2125171)
Supplement: Supplemental Material [file IANN_A_2125171_SM4283.zip › Files/IMRT2_Supplementary_Figure_legend_021522_YN[AU].docx]

**Supplementary Figure Legend**

Supplementary Figure 1. (A) Example of a conventional TBI irradiation method using a lead block in our hospital. (B) Representative radiation dose distribution of an IMRT-TBI for a 12 Gy prescription dose in the coronal (left), and axial view (right).
