# Supplementary figures and images for: Outcomes of allogeneic haematopoietic stem cell transplantation with intensity-modulated total body irradiation by helical tomotherapy: a 2-year prospective follow-up study
Source: Ann Med. 2022 Oct 17;54(1):2617–26. doi: 10.1080/07853890.2022.2125171 (PMC9624256; doi:10.1080/07853890.2022.2125171)

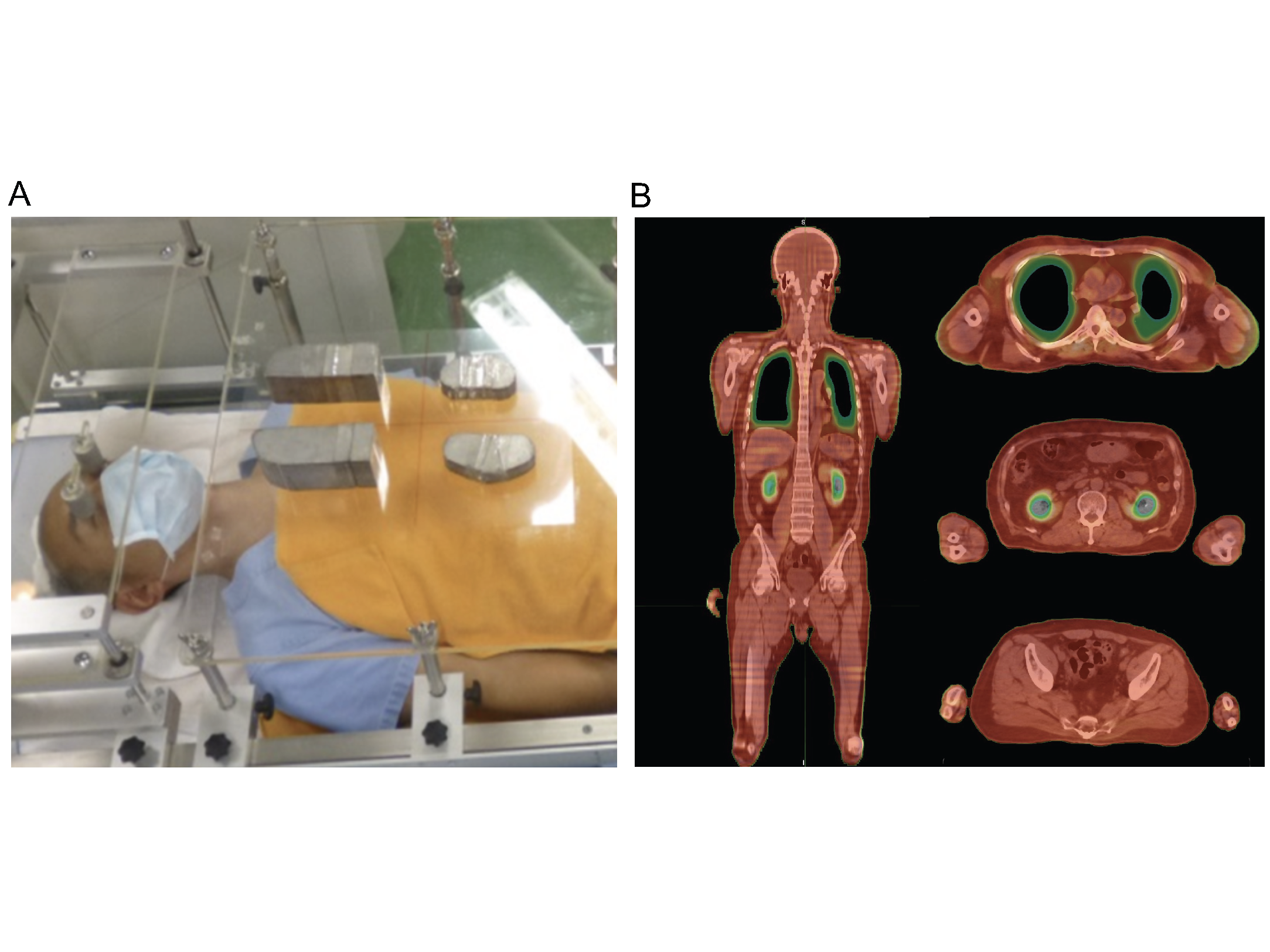

Supplement: Supplemental Material [file IANN_A_2125171_SM4283.zip › Files/IMRT2_Supplementary Figure1_021522[AU].tiff]
